# Wearable-Derived Heart Rate Variability Across the Menstrual Cycle, Hormonal Contraceptive Use, and Reproductive Life Stages in Females: A Living Systematic Review

**DOI:** 10.1007/s40279-025-02388-y

**Published:** 2026-01-17

**Authors:** Eline de Jager, Brian Caulfield, Evgenia Angelidi, Brian MacNamee,, Sinead Holden

**Affiliations:** 1 School of Public Health, Physiotherapy and Sport Science, University College Dublin, Dublin, Ireland; 2 Insight, Research Centre for Data Analytics, Dublin, Ireland; 3 Institute for Sport and Health, University College Dublin, Dublin, Ireland; 4 School of Computer Science, University College Dublin, Dublin, Ireland

## Abstract

**Background:**

Heart rate variability (HRV) reflects autonomic nervous system function and can now be continuously monitored in real-world settings using wearable technology. However, the influence of ovarian hormones on HRV remains unclear, underscoring the need to synthesize evidence across the female lifespan.

**Objective:**

To examine the association between ovarian hormone profiles and HRV measured by wearable/mobile devices.

**Design:**

Living systematic review.

**Data Sources:**

A comprehensive search was conducted in PubMed, Web of Science, IEEE Xplore, SPORTDiscus, and Embase from inception to December 2025. The search followed the Participant (females of any age), Exposure (ovarian hormone profiles), and Outcomes (HRV measured by wearable devices) framework, using a combination of MeSH terms and keyword adaptations. Quality was assessed for cohort studies using the Newcastle–Ottawa Scale.

**Eligibility Criteria:**

All studies were independently double screened by title, abstract, and full text. Studies were eligible if they examined differences in HRV measured by a wearable device, across the menstrual cycle in naturally menstruating females, in response to exogenous ovarian hormones, or across another ovarian hormone state such as menopause or pregnancy.

**Results:**

From 299 identified records, 16 studies were included. In naturally menstruating females, HRV was higher at the beginning of the cycle and lower toward the end, with differences in time-domain HRV ranging from 3 to 9%. Hormonal contraceptive users exhibited lower HRV, particularly in the late cycle. Additionally, HRV tended to decline after menopause with increasing age. The quality of evidence in this review was moderate (7/9). Variability in how menstrual cycle phases and menopausal status were classified across studies limited comparability and the ability to synthesize findings quantitatively.

**Conclusions:**

Wearable-derived HRV is associated with differences across the menstrual cycle, oral contraceptive use, and reproductive life stages. This should be considered when presenting HRV metrics to female users. This may improve the interpretation of data for female athletes, patients, or women who track their HRV.

**OSF Registration:**

DOI https://doi.org/10.17605/OSF.IO/S4RYW.

**Supplementary Information:**

The online version contains supplementary material available at 10.1007/s40279-025-02388-y.

## Key Findings

**Table:** 

Wearable-measured HRV varies across the menstrual cycle, with most studies reporting higher values at the start of the cycle compared with the end of the cycle.
Wearable devices tracking HRV reveal that hormonal contraceptives users generally show different HRV patterns compared with naturally menstruating women.
Few studies use gold-standard methods (e.g., hormonal assays or ovulation confirmation via LH/ultrasound) to verify menstrual phase or ovulatory status, limiting the precision of phase-based interpretations.
Methodological heterogeneity in HRV measurement protocols (e.g., device type, duration, timing, and HRV metrics) poses significant challenges for comparability and precludes meta-analysis.

## Introduction

Heart rate variability (HRV) is a noninvasive marker of autonomic nervous system function and refers to the variation in time intervals between consecutive heartbeats [1, 2]. This reflects a dynamic balance between parasympathetic and sympathetic activity. Higher HRV is associated with parasympathetic function, suggested to indicate relaxation and recovery, whereas lower HRV indicates sympathetic dominance, linked to physiological stress [3]**.**

Recent advancements in wearable technology, including improved sensor accuracy, photoplethysmography (PPG) techniques, and enhanced signal-processing algorithms, have expanded HRV monitoring beyond controlled laboratory settings [4]. These developments now allow individuals and athletes to track physiological responses in real-world settings [5] using a range of devices such as smartwatches, chest straps, smart rings, and fitness patches. Wearable technology offers a valuable opportunity to obtain daily, real-world HRV measurements in athletes, patients, or other female users. This provides more accurate insights into the interaction between hormonal fluctuations and autonomic regulation.

However, the reliability and validity of wearable devices to capture HRV depend on the underlying measurement method. Electrocardiography (ECG) remains the gold standard for HRV assessment, as it directly measures the electrical activity of the heart [2]. PPG, used in most wearables, instead detects blood volume changes at the skin surface to estimate HRV. While modern PPG-based devices show good agreement with ECG during resting or low-motion conditions [6], their accuracy decreases with movement, poor sensor contact, or irregular heart rhythms [7].

Despite the increasing use of HRV, existing research has been predominantly conducted on male populations [8, 9]. Sports science research has historically been male-dominated, resulting in a significant knowledge gap regarding female physiology [8, 10, 11]. Evidence suggests that HRV fluctuates across the menstrual cycle in naturally menstruating females [12]. A systematic review found higher HRV [12] during the follicular phase and lower HRV in the luteal phase, suggesting a state of decreased parasympathetic activity [13]. Moreover, while both endogenous hormones and hormonal contraceptive use are believed to be associated with changes in HRV [14], the current body of research lacks consistent and conclusive evidence regarding the nature and magnitude of these hormonal effects.

While HRV is increasingly measured in the real world with wearable devices [15, 16], it remains unclear whether these devices can capture differences in HRV across ovarian hormone profiles in real-world settings. In this regard, in situ wearable-derived measurements represent a promising approach for studying hormone-linked HRV variations in a real-world context.

However, it is also important to consider whether observations obtained via wearable devices align with those reported in controlled laboratory studies. Examining this consistency helps contextualize wearable-derived findings and assess the validity of real-world HRV measurements.

This systematic review aims to examine wearable-derived HRV across ovarian hormone profiles. Specifically, it evaluates the impact of the menstrual cycle in naturally menstruating females, assesses the impact of hormonal contraceptives, and explores how HRV changes across reproductive life stages, including reproductive age, perimenopausal, and postmenopausal women. Given the rapidly evolving nature of research in this field, a living systematic review approach is used to ensure continuous integration of emerging evidence. A living review is a type of systematic review that is continually updated as new studies become available, allowing the synthesis to remain current over time.

## Methods

This systematic review is reported according to the Preferred Reporting Items for Systematic Reviews and Meta-Analysis (PRISMA) statement guidelines [17], the PRISMA extension for living systematic reviews (PRISMA-LSR) guidelines [18], and the implementing Prisma in Exercise, Rehabilitation, Sport medicine and SporTs science (PERSiST) guidance [19]. The study was registered on Open Science Framework (OSF: S4RYW) before data extraction.

### Search Strategy

The literature search was performed using five databases: PubMed, Web of Science, IEEE Xplore, SPORTDiscus, and Embase. The search was based on the Participants, Exposure, and Outcome (PEO) framework. Search terms were used for females (Participants), ovarian hormone profiles (Exposure), and the outcome of wearable device-measured HRV (Outcome). The full search strategy with all the database-specific search strategies implemented in this study is included in the Supplementary Information. Search terms were identified using a combination of keyword searches and Medical Subject Headings (MeSH), or similar methods where available. The initial search was carried out on 17 October 2024, with the most recent search completed on 17 December 2025. The initial data extraction was carried out from October to December 2024. Additionally, references from included studies were reviewed to identify further relevant research.

### Living Mode Parameters

To ensure that the review remains up to date and incorporates newly available research, a living review approach is employed. Monthly searches with email notifications are enabled for all databases. These are then screened to identify any new potentially eligible studies. This process led to the inclusion of one additional article in January 2025, and an additional two articles were added in October 2025. Updates will be posted on OSF every 6 months, and if the new findings have an important impact on the review findings, an update of the review will be published. The living review will retire after 4 years, upon completion of the PhD research of the author.

### Study Selection and Eligibility

Covidence, a web-based collaboration software platform that streamlines the production of systematic and other literature reviews [20], was used for the selection process. Both title and abstract screening and full-text screening were conducted independently by two authors (E.D.J. and S.H.). The additional papers added in October 2025 were screened by two authors (E.D.J. and E.A.). Any disagreement was resolved collaboratively until a consensus was reached.

The review applied specific inclusion and exclusion criteria according to the PEO framework. Studies were included if they focused on females of any age and measured HRV either across different menstrual cycle phases, in individuals using exogenous hormones, or across different life stages such as premenopausal versus menopausal. Only original, peer-reviewed studies published in English were considered. Additionally, only studies that measured HRV using wearable or mobile devices outside a laboratory setting were included. Wearables and mobile health monitoring devices are electronic tools that people can comfortably wear on their bodies or use daily to monitor health metrics, including HRV. These devices are designed to be compact and unobtrusive, often taking the form of wristbands, smartwatches, chest straps, rings, wearable/wireless ECG devices, or mobile apps that use camera- or finger-based photoplethysmography (PPG) on smartphones.

Studies were excluded if they focused on cardiovascular metrics other than HRV or did not involve any hormonal profiles. Research that included only male participants or did not address hormonal variations in females was also excluded. Furthermore, studies that measured HRV using methods other than wearables or user-applied devices were not considered. Nonhuman studies, as well as reviews, editorials, case studies, or studies published in languages other than English, were not included in the review.

### Data Extraction

Data extraction was conducted independently by two reviewers (E.D.J. and E.A.) using a custom data extraction template in Excel. The following general information was extracted from the included studies: publication year, lead author, study aims, study design, inclusion criteria, and statistical analysis. Next, participant details were recorded, including number of participants, their characteristics (such as physical activity/sport level, age, and body mass index (BMI)), and the focus of the study (whether participants were naturally menstruating, using hormonal contraceptives, or in specific reproductive life stages). For each group, the specific menstrual cycle phases, hormonal phases, or reproductive life stages studied were identified, along with how these phases and stages were determined, the number of participants within each group, and the duration of participant monitoring. Regarding data collection, details about the methods used for ovarian hormone measurement were extracted, as well as the days on which data were collected and which specific hormones. Information on HRV metrics was noted, including the devices used for HRV assessment, measurement protocols, including the sensor type, time of day (e.g., morning on waking, overnight, spontaneous), body position (e.g., lying supine or seated), position of the wearable on the body, duration of the recording, and instructions before the recording. If daily questionnaires were included in the study, the content they recorded was also documented. Finally, the results and conclusions of each study were included. There were no disagreements between reviewers.

### Quality Assessment

The quality of the studies was assessed using the Newcastle–Ottawa Assessment Scale (NOS) for cohort studies [21]. The NOS evaluates studies on the basis of three criteria: selection of cohort groups, comparability of cohorts, and the ascertainment of the outcome of interest [21]. The NOS assigns a star rating in each domain, with a maximum of nine stars indicating the highest quality. The study quality assessment for all included studies was independently and separately performed by two authors (E.D.J. and E.A.). Any disagreements were resolved by discussion to reach a consensus between the two review authors, with a third review author acting as an arbiter if necessary.

### Data Synthesis

This systematic review examines how HRV responds to menstrual cycle phases. Owing to the anticipated heterogeneity in study designs, including ovarian hormone profiles, outcomes (measures of HRV), and devices, a qualitative synthesis of findings was undertaken. Findings were synthesized according to the three aims: (1) investigating HRV fluctuations across different phases of the menstrual cycle in naturally menstruating females, (2) examining the effects of hormonal contraceptives on HRV, with a focus on how different hormonal contraceptive methods influence HRV across hormonal cycle phases, and (3) exploring HRV response to hormonal fluctuations across different reproductive life stages, including reproductive age, pregnancy, and menopause.

## Results

The database searches yielded 299 records, with 61 duplicates automatically removed, leaving 239 unique studies for screening (for the full PRISMA flow diagram, see the Supplementary Information). One hundred eighty-five studies were excluded by title and abstract. Of the 54 studies that underwent full-text review, 16 [22–37] were included (Table 1).

**Table 1:** Study characteristics of all 16 publications used in this systematic review

Author (year of publication)	Study design	Sample size	Participant age, BMI	Participant sport/activity characteristics	Inclusion criteria	Ovarian hormone profiles	Comparisons	Wearable utilized
Ahokas et al. (2023) [22]	Prospective observational study One MC or 4 weeks in PU and CU	*n* = 42	Age 26.3 ± 4.8 years BMI 22.7 ± 2.5 kg/m^2^	Healthy, normal weight, trained females	Not pregnant/breastfeeding. No PCOS, endocrine disorders, or diseases affecting ovarian/ANS function. NM = regular MC > 6 months. PU = hormonal IUD, CU = BC pill/rings	NM used an ovulation kit (Sofi, Finland) 4–6 days before the expected ovulation NM & PU provided four fasted blood samples, and CU provided two. PU samples were taken ~ 7 days apart (range 5–9 days) Serum estradiol (E2), progesterone (P4), and luteinizing hormone (LH) levels were measured	NM (*n* = 19): bleeding, follicular phase, ovulation, luteal phase PU (*n* = 12): comparable phases CU (*n* = 11): active and inactive pill phases	Bodyguard 2 HRV monitor Kubios software for analysis. Nightly HRV, 4-h period. Daily diary: bedtime, recovery perception, alcohol use, MC/HC symptoms, open comments
Altini & Plews (2021) [23]	Prospective observational study At least five MCs	*n* = 639	Age 37 ± 12 years BMI 23.8 ± 3.2 kg/m^2^	Training occasionally to training daily	No specific inclusion criteria	Follicular and luteal phase estimation based solely on self-reported menses and predicted ovulation	Follicular and luteal phases	HRV4Training app, short-term, upon waking measurement. Annotations: Training info, alcohol intake, menstruation days, sickness
Alzueta et al. (2022) [24]	Prospective observational study One MC	*n* = 26	Age 24.4 ± 1.1 years	Healthy women	Age 18–35 years, regular ovulatory MCs (22–35 days) menses < 10 days. No PMS /PMDD. No medical conditions/contraceptive use	Commercial urine tests for luteinizing hormone (LH) were used. Starting 5 days before the expected ovulation date until 3 days after ovulation	Menses: 4 days (starting at onset) Ovulation: 2 days (from first positive urine test) Midluteal: 4 days (starting 6 days after positive LH test) Late luteal: 4 days (starting 4 days before menstruation)	Oura ring, 5-min intervals overnight. Daily diary: sleep, mood, readiness, physical symptoms
Alzueta et al. (2024) [25]	Prospective observational study At least one MC	*n* = 116	Young (18–35 years, reproductive age) Midlife (42–55 yeare, late reproductive to menopause transition)	Not specified	Age 18–35 years, regular MC (22–35 days) 42–56 years. Smoke < 4x/week. No PMS/ PMDD. No contraceptive use	The trend of distal skin temperature data collected via the Oura ring is used for MC reflection. Participants also recorded days of menses and used a commercial urine test for LH from 5 days before expected ovulation until 3 days after ovulation	Young (*n* = 64), midlife (*n* = 52) Menses: days 1–4 (day 1 = bleeding) Ovulation: confirmed positive LH, + next Mid-luteal: 4 days, starting 6 days after positive LH Late-luteal: starting 4 days before the next menses	Oura ring Gen2, HRV measured in 5-min intervals overnight. Daily diary: sleep, mood, symptoms, and days of menses
Andric et al. (2021) [26]	Nonrandomized experimental study One MC	*n* = 25	Age 20.5 ± 0.7 years BMI 21.57 ± 2.2 kg/m^2^	No more than an hour of sports per day for no more than 3 days a week	Regular MC	Phase estimation solely based on self-reported menses and days in the cycle	Day 14 MC = day 0 Bleeding Early follicular phase (day − 15 until − 6) Late follicular phase (day − 5 until day − 1) Mid-luteal phase (day + 5 until day + 9)	Polar RS800CX rest HRV sitting on ergometer for 5 min PolarProTrainer for analysis
Goodale et al. (2019) [27]	Prospective observational study Up to a year or until pregnant	*n* = 193	Age 33.02 ± 3.68 years BMI 22.7 ± 3.4 kg/m^2^	Not specified	Swiss women, aged 18–40 years. Regular MC (24–32 days), trying to conceive. Excluded if unable to confirm LH surge	Urinary luteinizing hormone test (Clearblue Advanced Fertility Monitor, SPD Swiss Precision Diagnostics GmbH) to determine the close of the fertile window from 5 days after onset of menses	Menstruation: first day menses, lasting 5 days Follicular phase: first-day post-menses, lasting through 6 days before ovulation Fertile window: 5 days before ovulation, lasting through ovulation Early luteal phase: 1 day after ovulation, lasting 1 week Late luteal phase: 8 days after ovulation, lasting until the day before menses	Ava bracelet (Ava AG). HRV was measured every 10 s overnight. Diary: activities, info about a day before
Gordon et al. (2023) [28]	Randomized, double-blind, cross-over study	*n* = 39	Age 24.6 ± 5.9 years BMI 23.7 ± 4.07 kg/m^2^	Recreationally active at least 3 × a week	BMI 18.5–29.9 kg/m^2^. No amenorrhea, no change of contraceptive < 6 months, not pregnant	Phases based on calendar dates and saliva estrogen levels. Basal temperature was used to confirm phase status	Naturally menstruating (*n* = 25), oral contraceptives (*n* = 8), intrauterine devices/vaginal rings (*n* = 6) Low hormone phase (beginning day 2–8) High hormone phase (beginning day 14–18)	Polar H10 (Polar Electro Oy, Kempele, Finland) strapped to the chest for 3 min resting HRV, the EliteHRV app for analysis. Diary: MC and temperature
Hamidovic et al. (2023) [29]	Prospective observational study One MC	*n* = 28	Age 26.5 ± 5.02 years BMI 25.55 ± 4.75 kg/m^2^	Not specified	Reproductive-age women with PMDD and healthy controls, no drugs/smoke. Including hormonal BC. Anovulatory cycles were removed	Daily LH testing and blood and saliva sample collection at eight time points in the MC	PMDD and healthy controls. Blood and saliva tests are done during early follicular, mid-follicular, periovulatory, early luteal, mid-luteal, and late luteal subphases	ZephyrTM BioHarness 3. 5-min HRV morning measure in a sitting position. DRSP for MC-related symptoms
Jasinski et al. (2024) [30]	Prospective observational study 22 months	*n* = 11,590	Age 34.88 ± 7.32 years BMI 25.56 ± 4.32 kg/m^2^	Not specified	Age 18 + years, use hormonal BC. Regular MC (21–35 days) bleeding < 7 days	Phase estimation solely based on self-reported menses	NM (*n* = 9968) or birth control pills (*n* = 1661)	WHOOP measured HRV during nonwake periods of primary sleep episodes. Diary: BC data, menstruation status
Kokts-Porietis et al. (2019) [31]	Prospective observational study 5 weeks	*n* = 7	Age 28.6 ± 8.4 years	Cycling/triathlon training as a primary activity Cross-training on bike > 3 h a week	No exogenous hormones, nonsmokers, 18–45 years. Regular MC (26–35 days) no amenorrhea/postmenopausal	Basal body temperature method of determining ovulation	Follicular phase (prior ovulation) Luteal phase (after ovulation)	HRV4Training app, 1 min measure before getting out of bed, supine position
Luo et al. (2025) [32]	Prospective observational study Two MC	*n* = 183	Age median 31 years BMI median 21.34 kg/m^2^	Not specified	Women aged 18–45 year with natural cycles, no current pregnancy or in past 6 months, not breastfeeding, no sleep disorders	Follicle monitoring ultrasound from day 8 to determine follicle, serum hormone assays for LH, estradiol, and progesterone until ovulation	Regular menstruating (*n* = 136) Irregular menstruating (*n* = 47)	Huawei Band 6 Pro worn at least 5 h each night. Daily surveys for symptoms
Markovic et al. (2024) [33]	Prospective observational study Up to 9 months	*n* = 1,613	Age 49.32 ± 12.8 years BMI 26.88 ± 5.44 kg/m^2^	Not specified	No specific inclusion criteria	Ava’s fertility detection algorithm (validated in clinical trials, with its accuracy on par with urine-based ovulation tests)	Not menstruating, menstruating, premenopausal Within menstruating (*n* = 179); menstrual, follicular, fertile window, early luteal and late luteal phase*	Ava Fertility Tracker (Ava AG) HRV measured every 10 sovernight
Pearson et al. (2025) [34]	Observational study 16 weeks	*n* = 24	Age 22 ± 3 years BMI 28.1 ± 4.2 kg/m^2^	Rugby athletes, highly trained national-level athletes	Athletes from the National Rugby League (NRL) Indigenous Women’s Academy	Daily LH testing and blood collection for estradiol and progesterone concentrations during phases determined by current consensus [38]	Naturally cycling (*n* = 11) (including eumenorrheic, oligomenorrhea, PCOS, anovulatory, and luteal phase deficiency) Hormonal contraception (*n* = 13)*	Oura ring. Daily survey for symptoms, sleep, and bleeding
Sanchez-Barajas et al. (2018) [35]	Cross-sectional observational study	*n* = 177	Age 50.47 ± 8.45 years BMI 28.42 ± 4.63 kg/m^2^	Not specified	Mexican women 45–57 years. Three groups according to STRAW criteria	FSH and serum morning cortisol were measured by ELISA commercial kits (ALPCO, USA)	Pre-menopause (*n* = 60), early post-menopause (*n* = 58), late post-menopause (*n* = 59)	RS800CX clock (Polar, Finland). 5-min measure in fasting condition after 10 min rest
Sherman et al. (2021) [36]	Prospective observational study 18 weeks	*n* = 36	Age 20 ± 1 years BMI 25.6 ± 3.4 kg/m^2^	Rowing athletes	No disease, regular MC, no amenorrhea/postmenopausal women, or been or become pregnant	Self-reported menses used for classification menses and no-menses phases	Menses versus no menses	HRV4Training. 1-min measure after 1-min stabilization upon arrival at the boathouse. Diary: menstruation phase, training load
Sims et al. (2021) [37]	Retrospective study	*n* = 4594	Age 33.5 ± 7.3 years BMI 24.5 ± 4.1 kg/m^2^	Total hours exercise/week 5.6 ± 4.1	Regular MC (25–35 days) or use of hormonal BC for > 9 months	Phase estimation solely based on self-reported menses and four times 25% of cycle days	NM (*n* = 3870), CU (*n* = 455), PU (*n* = 269)	WHOOP measured HRV during the last slow-wave cycle of sleep

*NM* naturally menstruating, *PU* progestin-only contraceptive users, *CU* combined hormonal contraceptive user, *BC* birth control, *MC* menstrual cycle, *DRSP* daily record of severity of problems

*No results presented from this group

### Study Characteristics

The included studies adopted various study designs: 12 prospective studies [22–25, 27, 29–34, 36], 1 cross-sectional observational study [35], 1 retrospective study [37], a randomized double-blind crossover study [28], and a nonrandomized experimental study [26].

Sample sizes ranged from 7 to 11,590 participants, with a total of 19,322 female participants across all studies (Table 1). Among the 16 studies, 15 primarily investigated HRV patterns across different phases of the menstrual cycle in naturally menstruating females [22–34, 36, 37], 6 studies explored the impact of hormonal contraceptives on HRV [22, 28, 30, 34, 36, 37], while 3 examined HRV responses to hormonal fluctuations across different reproductive life stages [25, 33, 35].

**Fig. 1:**
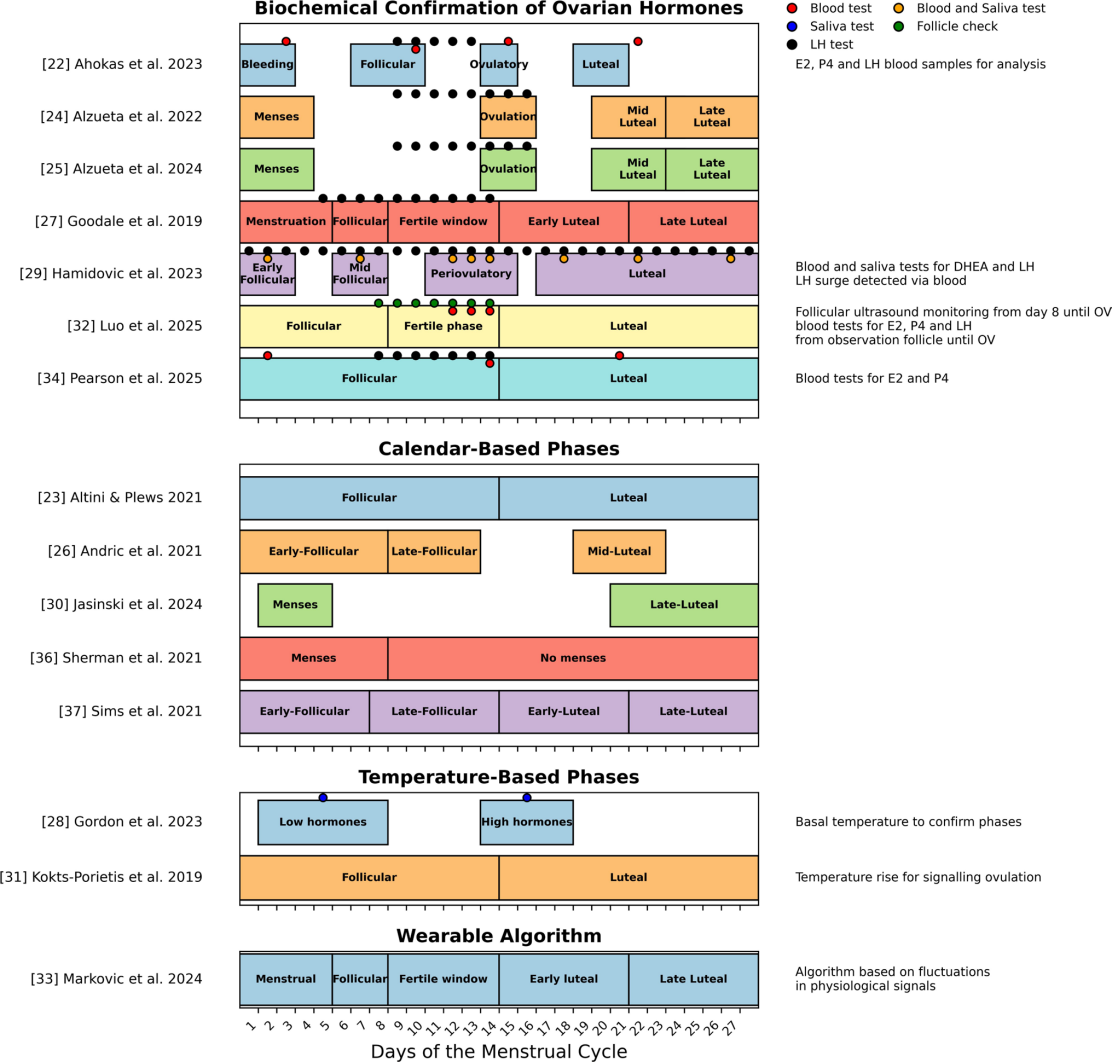
Visualization of the menstrual cycle phases as outlined in the studies included in this systematic review. The data are categorized by the method used to classify the menstrual cycle phases: biochemical confirmation of ovarian hormones, calendar-based tracking, or temperature-based monitoring. All data are normalized to a standard 28-day cycle, with actual phase durations varying according to the menstrual cycle lengths of the participants. *OV* ovulation, *E2* estrogen, *P4* progesterone, *LH* luteinizing hormone, *MC* menstrual cycle, *DHEA* dehydroepiandrosterone

Among the 15 studies analyzing HRV variations throughout the menstrual cycle, 7 utilized biochemical confirmation of ovarian hormones to determine cycle phases [22, 24, 25, 27, 29, 32, 34]. Two studies employed a temperature-based approach [28, 31], one used a wearable algorithm [33], while five relied on calendar-based phase estimation [23, 26, 30, 36, 37]. Of these, two studies predicted ovulation to be at the halfway point of the cycle and used that to split follicular and luteal phase [23, 37], while one used day 14 as the predicted ovulation day to split the two phases [26]. Of those using biochemical ovarian hormones confirmation, six detected the luteinizing hormone (LH) surge via LH tests to estimate the timing of ovulation [22, 24, 25, 27, 29, 34], while one study used follicle monitoring with ultrasound imaging to detect ovulation [32]. In general, the detection of LH surge/ovulation was used for the ovulation/fertile window and to identify mid-late luteal phase, 6–10 days post-ovulation, while in other cases, ovulation was used to split the follicular and luteal phases. The specific phase divisions can be found in Fig. 1.

**Fig. 2:**
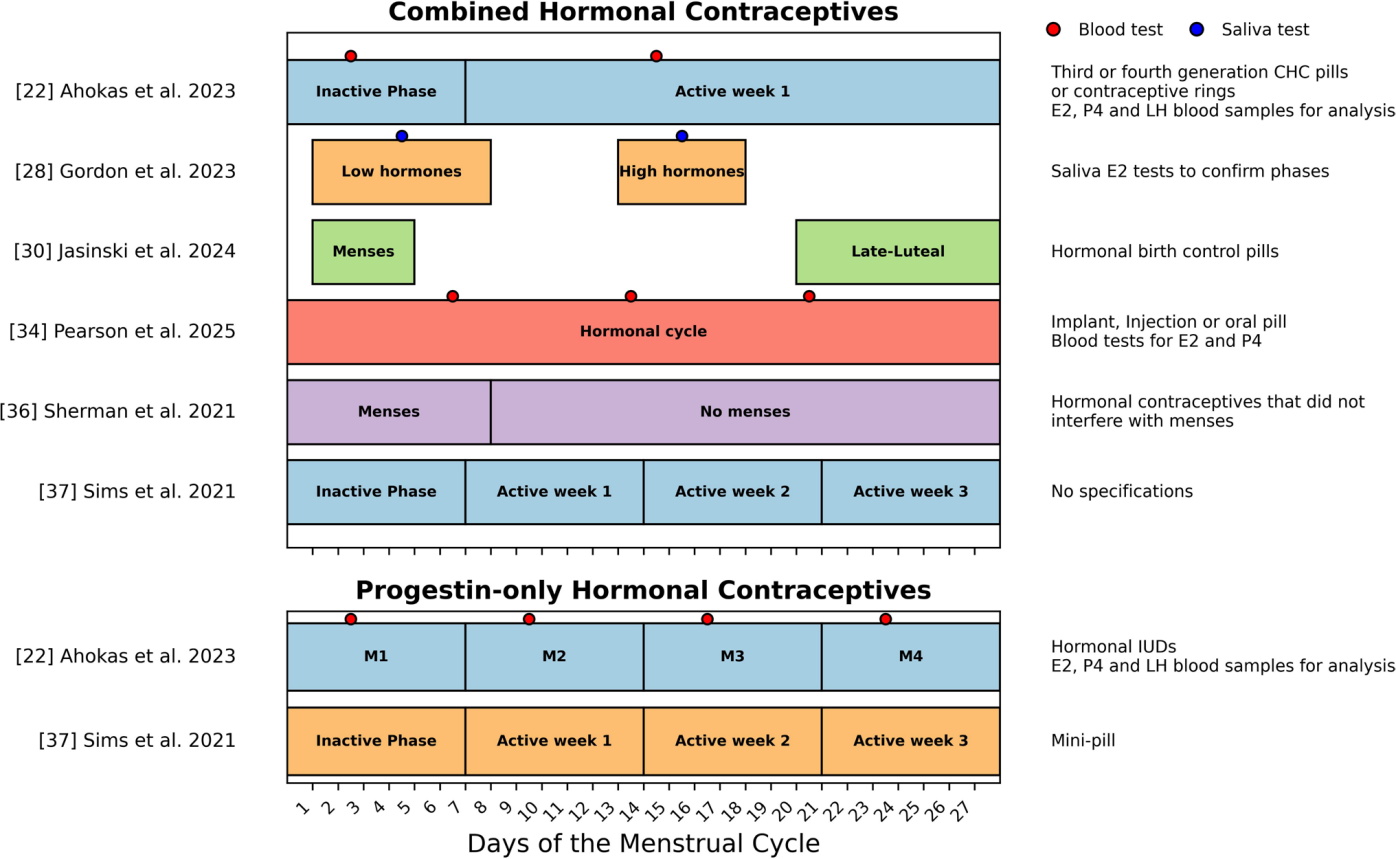
Visualization of the hormonal cycle phases as outlined in the studies included in this systematic review. The data are categorized according to combined and progestin-only hormonal contraceptive use. The specific contraceptives used per study are mentioned on the right. All data are normalized to a standard 28-day cycle, with actual phase durations varying according to the menstrual cycle lengths of the participants. *CHC* combined hormonal contraceptive, *IUD* intrauterine device, *E2* estrogen, *P4* progesterone, *LH*, luteinizing hormone

Five studies assessed hormone levels through different means. Four used blood tests, two measuring estrogen, progesterone, and LH levels [22, 32], one assessing estrogen and progesterone [34], and another assessing dehydroepiandrosterone (DHEA) and LH [29]. Additionally, one study assessed estrogen levels using a salivary test [28], and one assessed DHEA and LH using saliva tests [29]. The primary phases examined in the studies were the follicular and luteal phases. However, in all studies that did not validate the phases with biochemical confirmation of ovarian hormones, references were made to the day or the beginning or end of the menstrual cycle. A visual representation of the menstrual cycle and the hormonal phases and classification methods used in the studies is provided in Figs. 1 and 2 and the Supplementary Information.

### HRV Measurement

The included studies assessed a range of HRV metrics, including time-domain measures such as the root mean square of successive differences (RMSSD), and frequency-domain metrics. An overview of the metrics used can be found in Table 2.

**Table 2:** Overview of HRV metrics included in the systematic review. Definitions by Shaffer et al. [36]

HRV metric	Definition
RMSSD	Root mean square of successive RR interval differences. The RMSSD primarily reflects parasympathetic activity, with higher values indicating greater vagal modulation and lower values suggesting reduced parasympathetic influence [1]
SDNN	The standard deviation of NN intervals. The SDNN represents overall autonomic variability, capturing both sympathetic and parasympathetic contributions. Higher SDNN values indicate greater overall autonomic flexibility, while lower values may reflect increased physiological stress or reduced adaptability [1]
HF power	The absolute power of the high-frequency band (0.15–0.4 Hz)
LF power	The absolute power of the low-frequency band (0.04–0.15 Hz)
LF/HF ratio	The ratio of LF-to-HF power
pNN50	Percentage of successive RR intervals that differ by more than 50 ms
RMSSDcv	Daily fluctuations measured by the coefficient of variation of RMSSD
Ln(RMSSD)	Log-transformed daily RMSSD values
RMSSDamp	The mean value of RMSSD of 7 days centered on day 5 of the menstrual cycle

*RMSSD* root mean square of successive differences, RR interval time measured between two successive R waves (QRS complex) on an electrocardiogram (ECG), *SDNN* standard deviation of NN intervals, *NN* interval time measured between two consecutive normal R waves on an ECG, *HF* high frequency, *LF* low frequency

**Fig. 3:**
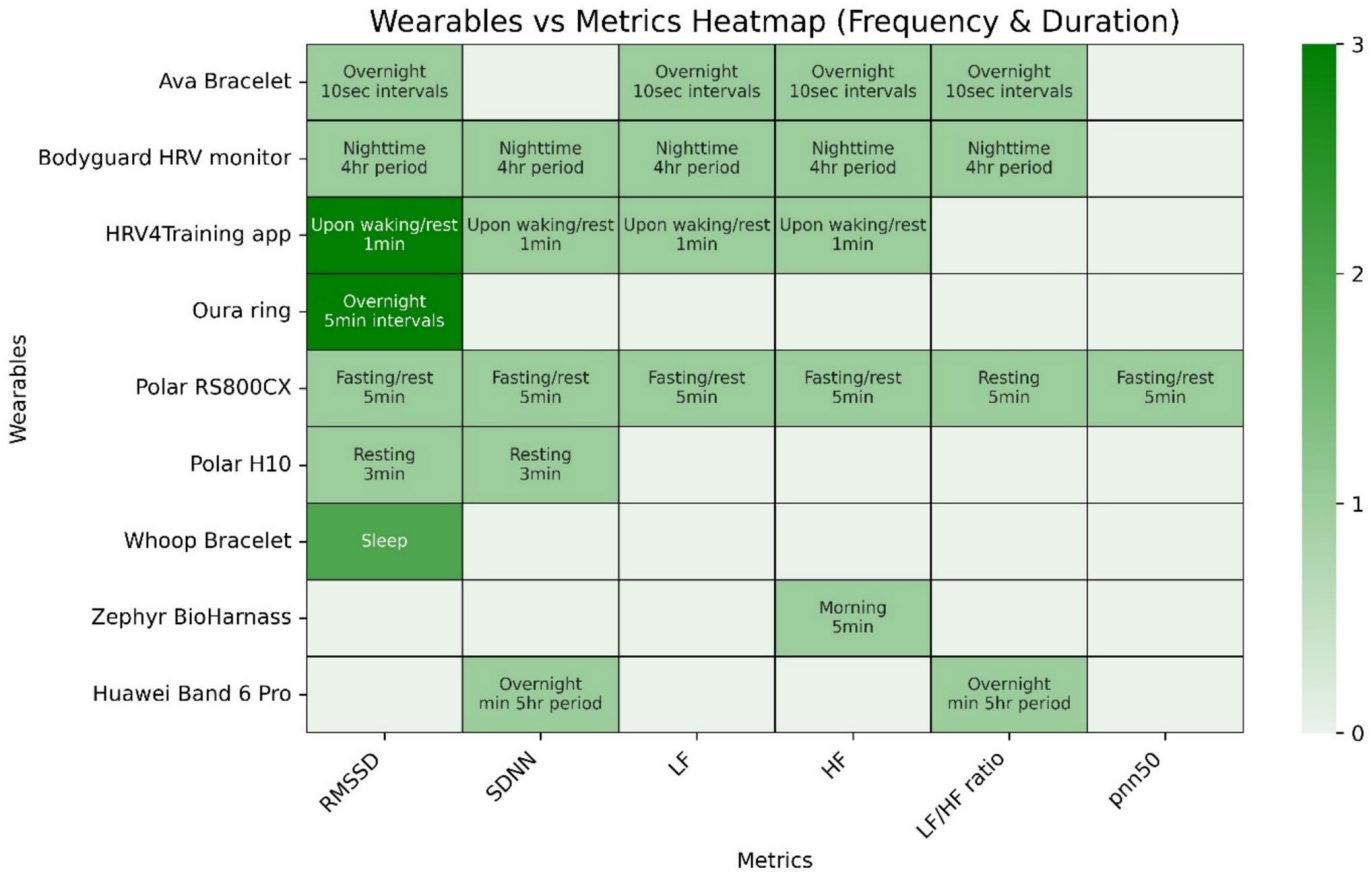
Heatmap of wearables and metrics used across studies, with color intensity indicating the number of studies. Measurement times and durations are provided in the text. An explanation of the metrics can be found in Table 2

Various wearable devices and smartphone applications were used to measure HRV. Three studies used the HRV4Training smartphone application [23, 31, 36], while two studies used the polar RS800CX watch [26, 30]. The Oura ring [24, 25, 34] was used by three studies. The Ava bracelet [27, 33] and the WHOOP were also each used in two studies [30, 37]. Other devices included the Bodyguard monitor [22], the Polar H10 chest strap [28], the Huawei Band 6 Pro [32], and the Zephyr harness [29]. Additionally, some studies used software applications for data extraction and analysis, such as PolarProTrainer [26], Kubios [22], or Elite HRV [28]. The specific HRV metrics captured by each device and the measurement specifications are summarized in Fig. 3, and the specific measurement protocols for each study can be found in Table 3.

**Table 3:** Measurement protocols used by all 16 publications in this systematic review

Author (year of publication)	Wearable used	Sensor type	Time of day	Duration recording	Body position	Position wearable on body	Measurement instructions	Data
Ahokas et al. (2023) [22]	Bodyguard 2	ECG	Overnight	4-h period, beginning 30 min after reported bedtime	Supine	Two spot electrodes, under right collarbone and on the left rib	Maintain habitual levels of training during measurement days	R–R intervals, Kubios Oy used to remove artifacts and obtain HRV variables
Altini & Plews (2021) [23]	HRV4Training application	PPG	Morning	1–5 min	Supine	Finger or placement of external sensor	Right after waking up while still lying down	Results provided by the app
Alzueta et al. (2022) [24]	Oura ring	PPG	Overnight	5-min intervals	Supine	Finger of the nondominant hand	No specific instructions	Synchronise with Oura app
Alzueta et al. (2024) [25]	Oura ring	PPG	Overnight	5-min intervals	Supine	Finger of the nondominant hand	No specific instructions	No specific instructions
Andric et al. (2021) [26]	Polar RS800CX	ECG	Day	5 min	Sitting on cycle ergometer	Chest	Sit quietly and breathe spontaneously for 5 min	Transferred to laptop via USB and analyzed in Polar ProTrainer
Goodale et al. (2019) [27]	Ava bracelet	PPG	Overnight	10-s intervals	Supine	Dorsal side of the wrist	No specific instructions	Synchronise with Ava app
Gordon et al. (2023) [28]	Polar H10	ECG	Day	30 min	Supine	Chest	8-h fast; lie supine for 30 min	Assessed through EliteHRV app
Hamidovic et al. (2023) [29]	Zephyr BioHarness	ECG	Morning	5 min	Sitting	Chest	No specific instructions	Assessed through Zephyr cloud
Jasinski et al. (2024) [30]	WHOOP	PPG	Overnight	Not given	Supine	Wrist	No specific instructions	Not given
Kokts-Porietis et al. (2019) [31]	HRV4Training application	PPG	Morning	1 min	Supine	Finger	Before getting out of bed, breathe normally, keep eyes closed, and stay relaxed	Results provided by the app
Luo et al. (2025) [32]	Huawei Band 6 Pro	PPG	Overnight	At least 5 h	Supine	Wrist	No specific instructions	Results calculated from R-R intervals
Markovic et al. (2024) [33]	Ava bracelet	PPG	Overnight	10-s intervals	Supine	Wrist	No specific instructions	Synchronise with Ava app
Pearson et al. (2025) [34]	Oura ring	PPG	Overnight	5-min intervals	Supine	Finger	No specific instructions	Synchronise with Oura app
Sanchez-Barajas et al. (2018) [35]	Polar RS800CX	ECG	Day	Not given	Not given	Chest	Fasting condition after 10 min rest	Not given
Sherman et al. (2021) [36]	HRV4Training application	PPG	Day	1 min	Seated in a chair with back support	Left index finger	Limit bodily movement and practice spontaneous breathing	Results provided by the app
Sims et al. (2021) [37]	WHOOP	PPG	Overnight	Not given	Supine	Wrist	No specific instructions	Not given

*PPG* photoplethysmography, *ECG* electrocardiogram

### Quality Assessment

Regarding the quality assessment, the median total score was 7 of 9 on the NOS (range 6–9). All studies were considered either truly or somewhat representative of the average female population, with securely recorded data and controls for multiple additional factors. However, most studies scored lower on the selection of a nonexposed cohort and follow-up duration, as most lacked either a nonexposed cohort or sufficient follow-up testing. The score of each study is shown in the Supplementary Information.

Specific menstrual cycle phases and methods used to define them are shown in Fig. 1. Specific hormonal cycle methods including specific contraceptives used are shown in Fig. 2

### HRV across the Menstrual Cycle in Naturally Menstruating Females

Fifteen studies (*n* = 15,273) investigated HRV changes across menstrual cycle phases in naturally menstruating females [22–34, 36, 37]. The most used HRV metrics were RMSSD and SDNN; information regarding these metrics can be found in Table 2.

Overall, ten studies examined RMSSD across the menstrual cycle. Eight found significantly lower RMSSD in the latter half of the cycle (assumed luteal phase). Altini and Plews [23] reported a 3.2% reduction in the second half of the cycle compared with the first. Jasinski et al. [30] observed a nonlinear association between RMSSD and cycle day, with highest HRV around day 5 (approximately + 3.6 ms above the average RMSSD) and lowest values around day 21 (− 3.2 ms below average). Similarly, Sims et al. [37] reported HRV values decreasing from about 70 ms in the first quarter of the cycle to below 64 ms in the last.

Ahokas et al. [25] found lower RMSSD in the late versus early stage of the cycle (*p* = 0.009). Sherman et al. [36] studied naturally menstruating and combined hormonal contraceptive (CHC) using rowers and found that the ln(RMSSD) coefficient of variation was increased in the first half of the cycle, particularly during menses (*p* < 0.001) [36]. Pearson et al. [34] reported similar findings in rugby athletes, with lower nocturnal RMSSD during the hormonally confirmed luteal phase compared with follicular (*p* < 0.001).

Kokts-Porietis et al. [31] observed a decline in RMSSD from the start to the end of the cycle, with a plateau from day 5 until approximately 5 days after ovulation (assumed luteal phase), and median increase in RMSSD variance of 150.06 ms^2^. Alzueta et al. [24] reported lower RMSSD in both the last (− 5.96 ms) and second-to-last (− 5.47 ms) quarters compared with the early phase, though these differences were not statistically significant owing to wide confidence intervals. Two studies found no significant differences in RMSSD across the cycle under resting conditions [26, 28]. However, during recovery from anaerobic exercise, RMSSD was higher in the early phase compared with later phases (6.29 ± 1.06 ms versus 5.20 ± 0.83 ms; *p* = 0.011) [26].

**Fig. 4:**
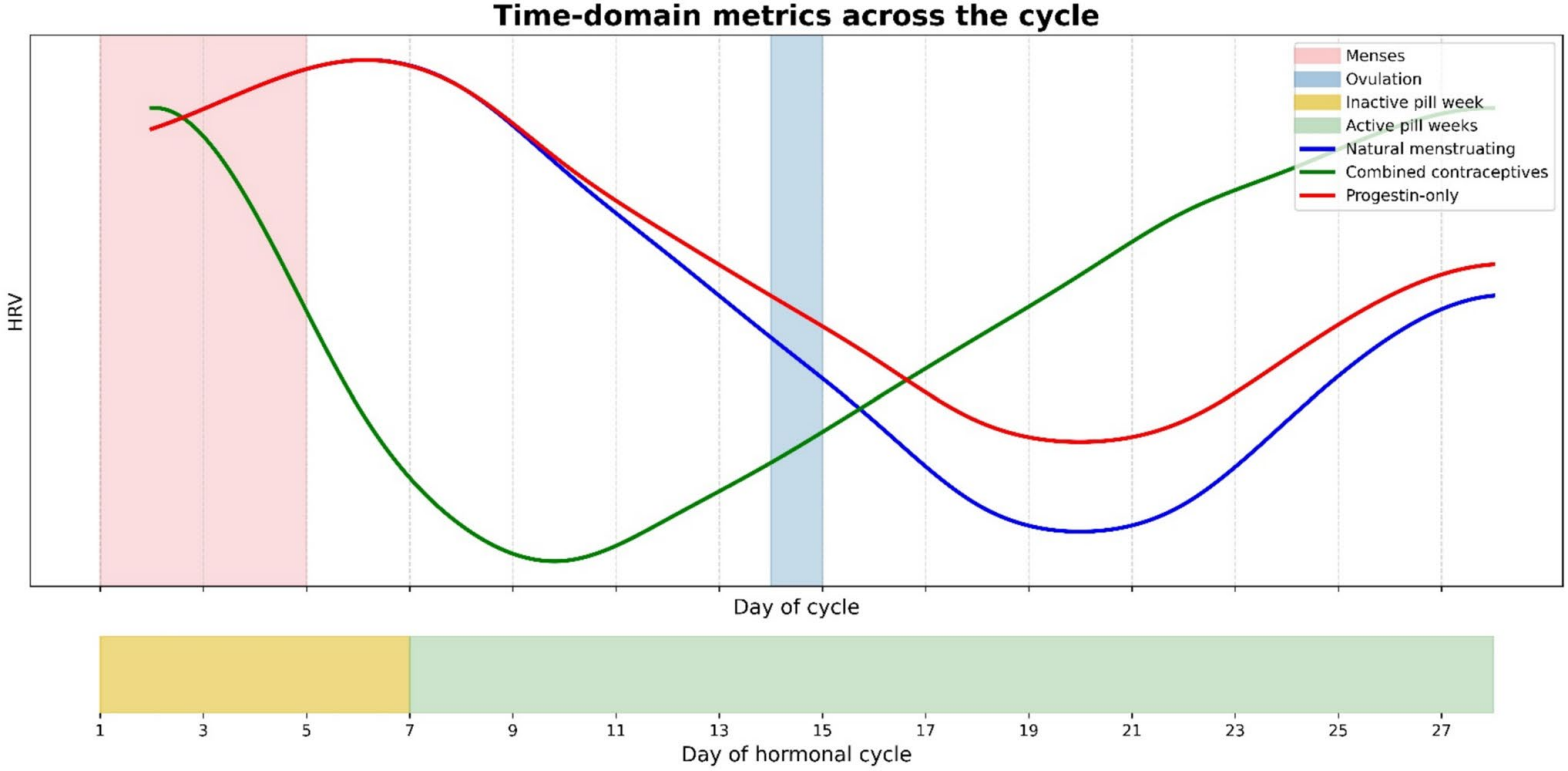
Conceptual visualization of HRV patterns (SDNN and RMSSD) across an idealized 28-day menstrual cycle and hormonal cycle for naturally menstruating individuals and users of hormonal contraceptives. Data are normalized to a 28-day cycle. This figure is illustrative only and does not represent actual data

SDNN showed similar trends in about two-thirds of studies. Ahokas et al. [22] showed higher values in the second quarter compared with the final quarter (*p* = 0.035). Kokts-Porietis et al. [31] showed an oscillatory pattern in daily SDNN medians, peaking on ovulation and again on days 4 and 11 after ovulation. Luo et al. [32] reported significantly lower ln(SDNN) during the fertile window and end of the cycle compared with the beginning, while Gordon et al. [28] found no significant differences across phases in naturally menstruating and CHC users combined. A visualization of the time-domain metrics across the cycle can be found in Fig. 4.

In two studies, hormonal fluctuations were examined in relation to HRV. Blood estrogen levels from bleeding to ovulation were negatively associated with RMSSD (*β* =  − 0.05; *p* < 0.001) and SDNN (*β* =  − 0.03; *p* = 0.007) [22]. The interaction between menstrual phase and estrogen levels was also significantly related to RMSSD (*β* =  − 0.05 to − 0.06; *p* ≤ 0.001) and SDNN (*β* =  − 0.04; *p* ≤ 0.002) [22]. Additionally, progesterone levels from blood and saliva were significantly related to HF power (*β* = 1.03; *p* ≤ 0.011) [29].

Frequency-domain metrics showed varying results. Hamidovic et al. [29] found significant differences in HF power between the early phase and peri-ovulatory days (*β* = 0.93; *p* < 0.001), as well as peri-ovulatory days and the following week (*β* =  − 0.70; *p* < 0.05) in their participant group including birth control users. In contrast, Kokts-Porietis et al. [31] showed stable HF medians throughout the cycle, with only a slight decrease around ovulation. For the low-frequency component, ln(LF) was higher at the start of the cycle compared with the end (3.62 ± 0.20 versus 3.39 ± 0.21; *p* = 0.009) [26].

Regarding the LF/HF ratio, Goodale et al. [27] found higher values in the week following menstruation (+ 0.11) and during the fertile window (+ 0.08) compared with the menstruation week. This ratio tended to decrease later in the cycle, with lower values in the final quarter compared with the first week (− 0.20) [27]. However, one study using biochemical confirmation of ovarian hormones found no significant phase differences [32].

### HRV and Hormonal Contraceptive Use

Six studies [22, 28, 30, 34, 36, 37] (*n* = 16,325) compared naturally menstruating females with hormonal contraceptive users, including those using hormonal intrauterine devices (IUDs), birth control pills, and contraceptive rings. In two of these studies [22, 37], hormonal contraceptives were further categorized into CHC and progestin-only contraceptives.

Jasinksi et al. [30] found that a group consisting of users of different types of hormonal birth control pills had significantly lower RMSSDamp compared with naturally menstruating females (− 0.51 ± 6.7 ms versus 4.65 ± 6.90 ms; *p* < 0.001), particularly in the second half of the menstrual cycle, with naturally cycling women maintaining higher values [30]. Sims et al. [37] observed that RMSSD in CHC users was initially elevated during the withdrawal bleed, followed by a significant decline at the onset of active pill use, before rising again throughout the active pill phase. Among CHC users (vaginal rings or pills) RMSSD (*p* = 0.014) was significantly higher during the low-estrogen phase compared with 7 days later [22]. Sherman et al. [36] studied naturally menstruating and CHC using rowers combined and found that the ln(RMSSD) coefficient of variation was increased during menses (*p* < 0.001) [36]. Women using progestin-only contraceptives exhibited a pattern like naturally cycling women, except at the very end of the cycle [37]. Among progestin-only users, RMSSD was significantly higher in the first active pill week compared with the second (*p* = 0.042)[22]. In CHC users, SDNN was significantly higher during the low-estrogen phase compared with 7 days later (*p* = 0.038) [22]. Additionally, in progestin-only users, SDNN was higher in the first active pill week compared with the second and third active weeks (*p* = 0.021–0.030) [22]. Gordon et al. [28] found no significant differences in SDNN and RMSSD across phases in naturally menstruating and CHC users combined. A visualization of the time-domain metrics across the cycle can be found in Fig. 4.

Regarding frequency-domain metrics, Ahokas et al. [22] reported that HF power was significantly higher in CHC users during the low-estrogen phase compared with 7 days later (*p* = 0.025). Similarly, in progestin-only users, HF power was elevated in the first active pill week compared with the second (*p* = 0.049) [22]. LF power in progestin-only users was significantly higher in the first active pill week compared with the second and third active weeks (*p* = 0.004–0.021). Additionally, LF power was elevated during the inactive pill week compared with the last active pill week (*p* < 0.001) [22]. No significant differences were found in the LF/HF ratio among CHC users; however, there was a trend (*p* = 0.07) toward a lower LF/HF ratio from the inactive phase to the second week of the active phase [22].

Ahokas et al. [22] found significant differences in the second week of the cycle for progestin-only contraceptive users (*p* = 0.042), as well as in the bleeding phase for the CHC users (*p* = 0.042) compared with the other hormonal cycle phases [22], when HRV metrics were adjusted for estrogen, LH, and progesterone levels.

### HRV in Reproductive Life Stages

Three studies (*n* = 1906 females) examined HRV in pregnant, premenopausal, menopausal, and postmenopausal groups [25, 33, 35]. To classify these stages, the terminology provided by Elliot-Sale was used to ensure methodological consistency: perimenopause as the time around the occurrence of menopause, menopause as the time when menstruation ceases, and post-menopause as the time after which a woman has experienced 12 consecutive months of amenorrhea [38].

Alzueta et al. [25] found that RMSSD was significantly lower in midlife females (ages 42–56 years) compared with younger, naturally menstruating females (ages 18–35 years), as measured using the Oura ring (*p* < 0.01) [25]. Sanchez-Barajas et al. [35] observed that pNN50 was highest in the premenopausal, naturally menstruating group (4.3 ± 6.2) compared with the early postmenopausal (< 5 years since last menstruation) and late postmenopausal (> 5 years since last menstruation) groups (1.6 ± 1.7 versus 2.4 ± 2.5, respectively; *p* = 0.001). However, no significant differences were found for RMSSD and SDNN in these groups.

LF power was elevated in both early (0.04 ± 0.02) and late (0.03 ± 0.01) postmenopausal females compared with premenopausal females (0.02 ± 0.01; *p* = 0.001) [35]. HF power was notably higher in the early postmenopausal group (0.09 ± 0.09; *p* = 0.042), whereas no other significant differences were reported in HRV metrics such as the LF/HF index [35]. In contrast, Markovic et al. [33] reported no difference in LF/HF ratio across groups (perimenopausal *β*-coefficient = 0.02; *p* = 1 versus postmenopausal and pregnant females, and menstruating females showing a *β*-coefficient =  − 0.05; *p* = 1 relative to the same group) [33].

## Discussion

This systematic review assessed how heart rate variability (HRV) changes across the menstrual cycle, hormonal contraceptive use, and various reproductive life stages, using data derived from wearable devices. The majority of studies reported that, in naturally menstruating females, HRV (RMSSD and SDNN) was generally higher early in the cycle, and generally declined at the end of the cycle in the assumed luteal phase. Nonetheless, some studies found no significant change, with frequency-domain metrics being more heterogeneous. Among hormonal contraceptive users, HRV patterns differed by contraceptive type. Combined hormonal contraceptive users typically showed reduced HRV following the onset of active pill use, with some recovery later in the cycle, while progestin-only users exhibited HRV patterns more like naturally cycling individuals, though with higher values toward the end of the cycle. Across reproductive life stages, HRV tended to be lower in midlife and postmenopausal individuals compared with younger, menstruating women, though findings varied by HRV metric. While these findings align with known autonomic patterns previously observed across menstrual cycle phases in controlled laboratory settings, notable inconsistencies across wearable-based studies highlight the need for more standardized protocols and stronger methodological reporting.

Wearable-derived HRV patterns largely mirrored those observed in laboratory-based settings, where higher parasympathetic activity (reflected in increased RMSSD and SDNN) is typically observed during the follicular phase, with a sympathetic shift during the luteal phase [12]. This similarity supports the ecological validity of wearable devices for detecting autonomic variation observed across the menstrual cycle in real-world settings [39].

HRV reflects the dynamic interplay between sympathetic and parasympathetic activity and serves as an indirect marker of autonomic regulation and physiological adaptability [1]. Higher HRV, particularly in time-domain measures such as RMSSD, generally indicates stronger parasympathetic (vagal) modulation and greater autonomic flexibility, while lower HRV often reflects sympathetic predominance or reduced vagal tone [1]. Because autonomic balance influences cardiovascular function, stress resilience, sleep quality, and recovery capacity, HRV is increasingly used as a noninvasive marker of both overall health and training readiness [40]. In athletes, HRV is sensitive to training load and cumulative stress, typically decreasing after intense exercise or inadequate recovery [41, 42]. However, its utility for prescribing or adjusting training remains debated. HRV responses are highly individual and influenced by factors such as sleep, hydration, illness, and hormonal functions [43], and short-term reductions may reflect normal adaptation rather than maladaptation.

Importantly, HRV’s interpretive value depends on measurement validity and protocol consistency. Time-domain indices, such as RMSSD, show good reliability and can be derived from short recordings, whereas frequency-domain measures require longer, stable recordings and display greater variability [1]. Device choice also matters: ECG-based chest straps provide more accurate R–R intervals, while PPG-based wearables are more susceptible to motion artifacts and pulse-transit variability, reducing reliability, particularly for frequency-domain metrics [1]. Consequently, longitudinal trends collected under consistent conditions are more informative than isolated measurements or comparisons with normative data. Overall, HRV offers valuable insight into autonomic dynamics when interpreted longitudinally, but should complement, rather than dictate, training or clinical decisions. Ultimately, HRV remains a proxy of neural activity, and its clinical and performance relevance depends on consistent measurement protocols and individual context [40, 44, 45].

Regarding the magnitude of differences detected, studies demonstrated changes of 3% to 20% across the cycle and even higher for reproductive life stages. Previous research has suggested that a change of approximately 3% in resting HRV may be meaningful, on the basis of the magnitude of change observed divided by the typical error [42]. Altini and Plews [23] reported that menstrual cycle effects were larger than those of other known factors, and above the smallest worthwhile change.

A key focus of this review was to assess whether wearable devices show similar findings in the real world to those observed in controlled laboratory settings. However, differences in device type, processing algorithms, and measurement protocols induce heterogeneity and preclude the pooling of data in meta-analysis. Differences including choice of HRV metrics (e.g., time-domain versus frequency-domain), device type (e.g., PPG-based versus ECG-based), timing (morning rest, nocturnal or daytime activity), recording duration (short-term versus long-term), and participant characteristics such as physical fitness, sleep quality, and comorbidities can significantly influence outcomes [1, 46–48]. For example, daytime measurements are more susceptible to external stimuli and sympathetic activation due to movement, stress, and environmental noise [49]. Additionally, frequency-domain measures such as LF and HF may be more affected by respiratory rate and require longer, artifact-free recordings than time-domain measures [40].

Most studies included in this review employed short or ultra-short HRV recordings typical of commercial wearables. However, frequency-domain analysis requires stationary recordings lasting at least ten times longer than the period of the slowest signal oscillations [47]. A minimum of 4 min is necessary to reliably assess time-domain metrics such as RMSSD and SDNN, while at least 5 min is recommended for valid frequency-domain HRV metrics such as LF, HF, and LF/HF ratio [50]. Some evidence suggests that time-domain measures are more reliable than frequency-domain and nonlinear metrics [50, 51], likely because they are less affected by breathing rate variability and noise, which are common challenges in wearable use during daily life. This poses a challenge for studies using ultra-short recordings common in commercial wearables. In practice, some commercial devices such as WHOOP, Oura, and Polar H10 report only time-domain metrics, as frequency-domain analysis cannot be accessed directly without raw data export. Although devices such as WHOOP and Oura collect HRV overnight, they still rely on relatively short measurement intervals, like the Polar H10 (Fig. 3). Given these constraints, time-domain metrics are generally considered sufficient for short-term recordings.

Another important consideration is that few studies explicitly accounted for confounding factors known to influence HRV. Variables such as training load, psychological stress, alcohol intake, and sleep quality were rarely measured in a standardized manner or constantly reported. Given that these factors can independently alter autonomic activity [43], their omission limits the ability to attribute HRV changes solely to hormonal fluctuations.

### HRV across the Menstrual Cycle in Naturally Menstruating Females

The majority of studies included were based on self-report measures, which means that menstrual cycle phases were estimated rather than measured. Studies employing hormonal verification more consistently detected HRV changes across the cycle [22, 24, 28, 29, 32, 34]. Two studies directly measured blood hormone levels and statistically correlated these with HRV outcomes [22, 29]. Hamidovic et al. [29] found a positive correlation between progesterone and HRV during the early start of the menstrual cycle; however, this correlation disappeared around ovulation. This suggests that factors other than rising progesterone levels contribute to the observed drop in HF-HRV around ovulation. Furthermore, Ahokas et al. [22] reported notable individual differences in estrogen concentrations, even among ovulating participants. Together, these findings indicate that self-reported or ovulation-test-based phase identification is insufficient to capture true hormonal variability. Misclassification of menstrual phases likely contributes to the inconsistent HRV patterns reported across studies, underscoring the need for repeated biochemical validation to accurately interpret hormone–autonomic interactions.

### HRV and Hormonal Contraceptive Use

Combined oral contraceptive users showed reduced HRV compared with naturally menstruating females [30], particularly after the start of the active pill phase [37]. This reduction in HRV may be related to the absence of the progesterone peak that typically occurs during the natural cycle. However, such changes may be overlooked in studies that collect HRV data only at a few broad time points rather than on a daily basis [14, 52]. Without frequent, high-resolution tracking, such as daily measurements, short-term fluctuations and temporary dips can easily go unnoticed. Two studies presented combined results for naturally menstruating and CHC users, which limits interpretability [28, 36].

In contrast, progestin-only users displayed HRV patterns like naturally menstruating females but with relatively higher values toward the end of the cycle [37]. These results suggest that different hormonal formulations exert unique effects on autonomic regulation. Additionally, it is important to mention that, within the two groups, viz. combined hormonal contraceptive users and progestin-only contraceptive users, differences were observed in the specific contraceptives used and in the classification of hormonal phases, as shown in Fig. 2.

### HRV in Reproductive Life Stages

Consistent with prior findings, HRV appeared to decline with advanced age. This review suggests that premenopausal women generally show higher parasympathetic modulation compared with peri- or postmenopausal individuals. While this pattern aligns with previous literature suggesting higher vagal tone during phases or life stages characterized by higher estrogen levels [35, 53, 54], it is important to consider that HRV naturally decreases with age in both sexes [55–57], making it challenging to separate hormonal influences from age-related effects. Some evidence even suggests that age itself may be a stronger determinant of HRV decline than hormonal status alone [55]. Notably, the studies included in this review used varying criteria to define menopausal status: two studies classified participants solely on the basis of age [25, 33], while one study combined age with a criterion of 5 years since the last menstrual cycle [35], following the methodological recommendations of Elliot-Sale et al. [38]. This variability in classification could contribute to inconsistencies in findings and highlight the need for clear, standardized definitions in the future.

Additionally, while time-domain metrics such as RMSSD and SDNN tended to decrease with age, some frequency-domain indices (e.g., HF power) were elevated post-menopause [13, 58–60]. These inconsistencies may partly be explained by methodological differences across studies, including the timing of HRV measurements (e.g., resting morning values versus 24-h continuous recordings) and the type of sensor used (e.g., ECG versus PPG-based wearables).

### Strengths and Limitations

This review’s strengths include its focus on wearable-derived HRV data and its exploration of hormonal influences across the reproductive lifespan in real-world conditions. However, several limitations should be considered. First, limiting the review to English-language publications may have excluded relevant non-English research. Second, only comparative studies were included, excluding single-group observational studies. While this enhanced the focus on comparative outcomes, it may have overlooked valuable descriptive data that could enrich the understanding of HRV patterns across different hormonal stages.

Third, substantial methodological heterogeneity across studies limited direct comparisons and precluded a meta-analysis. Key sources of variability included differences in HRV metrics, device types, recording durations, and definitions of menstrual cycle phases and menopausal status. Time-domain and frequency-domain measures assess different physiological aspects [1], which complicates data pooling. Device types varied between ECG and PPG-based wearables, with PPG producing less precise interbeat interval estimates owing to motion artifacts, pulse transit variability, and shorter effective sampling windows, affecting comparability. Recording durations ranged from ultra-short intervals (10 s to 1 min) to overnight or multi-hour measurements, with shorter recordings being more susceptible to noise and insufficient for reliable frequency-domain analysis [1].

However, the most important source of heterogeneity was inconsistent classification of menstrual cycle phases and menopausal status, which directly affects the physiological context of HRV measurements. Differences in how phases were defined, ranging from biochemical confirmation of ovarian hormones to self-reported cycle days, fundamentally influence the interpretation of HRV results. This variation in phase identification, combined with divergent HRV metrics and device types, had the greatest impact on between-study comparability, ultimately making meta-analysis unfeasible.

Finally, as mentioned above, most of the included studies did not adhere to the methodological considerations outlined for research in sport and exercise science [38]. Many studies relied on calendar-based approaches, in which menstrual cycle phases were estimated rather than directly measured [61]. To account for this limitation, results were reported according to the specific days of the cycle rather than by phase. However, it should be noted that this approach may still be misleading, as estimated phases do not always correspond precisely to physiological changes. A similar issue applies to the classification of menopausal status, where some studies relied solely on age instead of combining age with years since the last menstruation. Additionally, for consistency and visual clarity, figures in this review normalize data to a 28-day cycle, allowing graphical comparison while acknowledging that true cycle length and phase duration differ across studies and individuals.

### Future Research Directions

Future research should combine calendar-based cycle tracking with biochemical confirmation of ovarian hormones validation, using urinary LH tests and serum estradiol and progesterone measurements to improve menstrual phase accuracy [62, 63]. Research involving female participants should also move beyond broad assumptions, explicitly accounting for menstrual status, contraceptive use, and menopausal stage, rather than grouping all women together or using vague classifications [38]. Adopting standardized classification frameworks [64] would improve consistency and comparability across studies.

Longitudinal HRV measurements across the reproductive lifespan remain limited, primarily owing to feasibility challenges. However, this work is important as hormonal changes are highly individual and influenced by life events such as pregnancy, contraceptive use, and menopause, further complicating HRV patterns. Future studies should also examine how lifestyle factors such as sleep, stress, and physical activity interact with hormonal regulation of HRV, as these likely moderate autonomic responses at different hormonal stages.

## Conclusions

From this review, it was found that HRV varies meaningfully across menstrual cycle phases, hormonal contraceptive use, and reproductive life stages, and can be captured using wearable devices in real-world conditions. These findings emphasize the need to contextualize HRV data within the framework of menstrual cycle phase and reproductive stage-related hormonal fluctuations when interpreting metrics in female users. Standardized research approaches and integrating hormone assays into study designs will be essential to fully realize the promise of wearable-based HRV monitoring in female athletes.

## Supplementary Information

Below is the link to the electronic supplementary material.

Supplementary file1 (PDF 371 KB)

## Declarations

### Availability of Data and Material

The data generated or analyzed during this study are included in this published article and its Supplementary Information.
