# Supplementary material for: Wearable-Derived Heart Rate Variability Across the Menstrual Cycle, Hormonal Contraceptive Use, and Reproductive Life Stages in Females: A Living Systematic Review
Source: Sports Med. 2026 Jan 17;56(5):1153–73. doi: 10.1007/s40279-025-02388-y (PMC13198475; doi:10.1007/s40279-025-02388-y)
Supplement: Supplementary file 1 — Supplementary file1 (PDF 371 KB) [file 40279_2025_2388_MOESM1_ESM.pdf]

Supplementary Material

Sports Medicine

Wearable-derived Heart Rate Variability Across the Menstrual Cycle, Hormonal Contraceptive Use, and Reproductive Life Stages in Females: A Living Systematic Review

Wearable-derived Heart Rate Variability in Females

Eline de Jager[1,3], Brian Caulfield[1,2], Evgenia Angelidi[1,2], Brian MacNamee,[2,4], Sinead Holden[1,3]

[1] School of Public Health, Physiotherapy and Sport Science, University College Dublin, Dublin, Ireland.

[2] Insight, Research Centre for Data Analytics, Dublin, Ireland.

[3] Institute for Sport and Health, University College Dublin, Dublin, Ireland.

[4] School of Computer Science, University College Dublin

Detailed Search terms by database

Pubmed

((("Women" [MeSH] OR "Female" [MeSH] OR women OR woman OR female*) AND ("Menstruation" [MeSH] OR "Menstrual cycle" [MeSH] OR "Gonadal Steroid Hormones"[MeSH] OR "Menopause"[MeSH] OR "Contraceptives, Oral" [MeSH] OR "Hormonal Contraception"[MeSH] OR "Ovarian hormone*" OR "Reproductive hormone*" OR "Follicular phase" OR "Luteal phase" OR "Ovulatory phase" OR Menses OR Premenstrual OR Ovulation OR Ovulate OR Ovulatory OR Menstruat* OR Menarche OR "menstrual" OR Catamenia OR Fertil* OR Menopaus* OR "Oral contraceptive" OR "Hormonal contraceptive*" OR Estrogen OR progesterone OR "LH surge" OR "Luteinizing hormone surge")) AND ("Heart rate variability" OR HRV OR "Heart rate variabilities" OR "Autonomic nervous system" [MeSH] OR "Parasympathetic nervous system" [MeSH] OR "Sympathetic nervous system" OR "Parasympathetic activity" OR "Sympathetic activity" OR "Cardiac autonomic function" OR "Heart period variability" OR "Cardiac autonomic regulation" OR "Vagal tone" OR "Sympathovagal balance" OR "Autonomic imbalance" OR "Heart rate" [MeSH] OR "R-R interval")) AND ("Wearable Electronic Devices" [MeSH] OR Photoplethysmography [MeSH] OR Wearable* OR "Smart band*" OR "Heart rate monitor" OR "HR monitor" OR "Smart watch*" OR Wristband OR (device* OR sensor OR sensors OR "sensing") OR "Polar" OR "Garmin" OR "Oura" OR PPG OR Photoplethysmography OR "chest strap*" OR "heart rate sensor" OR "Apple Watch" or Fitbit OR Whoop)

Web of Science

(((ALL=(women OR woman OR female*)) AND ALL=("Menstrual cycle" OR "Gonadal Steroid Hormones" OR "Ovarian hormone*" OR "Reproductive hormone*" OR "Follicular phase" OR "Luteal phase" OR "Ovulatory phase" OR Menses OR Premenstrual OR Ovulation OR Ovulate OR Ovulatory OR Menstruat* OR Menarche OR "menstrual" OR Catamenia OR Fertil* OR Menopaus* OR "Oral contraceptive" OR "Hormonal contraceptive*" OR Estrogen OR progesterone OR "LH surge" OR "Luteinizing hormone surge")) AND ALL=(“Heart rate variability” OR HRV OR “Heart rate variabilities” OR “Autonomic nervous system” OR “Parasympathetic nervous system” OR “Sympathetic nervous system” OR “Parasympathetic activity” OR “Sympathetic activity” OR “Cardiac autonomic function” OR “Heart period variability” OR “Cardiac autonomic regulation” OR “Vagal tone” OR “Sympathovagal balance” OR “Autonomic imbalance” OR “Heart rate” OR “R-R interval”)) AND ALL=("Wearable Electronic Devices" OR Wearable* OR "Smart band*" OR "Heart rate monitor" OR "HR monitor" OR "Smart watch*" OR Wristband OR (device* OR sensor OR sensors OR “sensing”) OR "Polar" OR "Garmin" OR "Oura" OR PPG OR Photoplethysmography OR "chest strap*" OR "heart rate sensor" OR "Apple Watch" or Fitbit OR Whoop)

SPORTDiscus

(women OR woman OR female*) AND ("Menstrual cycle" OR "Gonadal Steroid Hormones" OR "Ovarian hormone*" OR "Reproductive hormone*" OR "Follicular phase" OR "Luteal phase" OR "Ovulatory phase" OR Menses OR Premenstrual OR Ovulation OR Ovulate OR Ovulatory OR Menstruat* OR Menarche OR "menstrual" OR Catamenia OR Fertil* OR Menopaus* OR "Oral contraceptive" OR "Hormonal contraceptive*" OR Estrogen OR progesterone OR "LH surge" OR "Luteinizing hormone surge") AND (=(“Heart rate variability” OR HRV OR “Heart rate variabilities” OR “Autonomic nervous system” OR “Parasympathetic nervous system” OR “Sympathetic nervous system” OR “Parasympathetic activity” OR “Sympathetic activity” OR “Cardiac autonomic function” OR “Heart period variability” OR “Cardiac autonomic regulation” OR “Vagal tone” OR

“Sympathovagal balance” OR “Autonomic imbalance” OR “Heart rate” OR “R-R interval”) AND (=("Wearable Electronic Devices" OR Wearable* OR "Smart band*" OR "Heart rate monitor" OR "HR monitor" OR "Smart watch*" OR Wristband OR (device* OR sensor OR sensors OR “sensing”) OR "Polar" OR "Garmin" OR "Oura" OR PPG OR Photoplethysmography OR "chest strap*" OR "heart rate sensor" OR "Apple Watch" or Fitbit OR Whoop)

IEEE Xplore

(women OR woman OR female) AND ("Menstrual cycle" OR "Gonadal Steroid Hormones" OR "Ovarian hormone*" OR "Reproductive hormone" OR "Follicular phase" OR "Luteal phase" OR "Ovulatory phase" OR Menses OR Premenstrual OR Ovulation OR Ovulate OR Ovulatory OR Menstruation OR Menarche OR "menstrual" OR Catamenia OR Fertility OR Menopause OR "Oral contraceptive" OR "Hormonal contraceptive" OR Estrogen OR progesterone OR "LH surge" OR "Luteinizing hormone surge") AND ("Heart rate variability” OR HRV OR “Heart rate variabilities” OR “Autonomic nervous system” OR “Parasympathetic nervous system” OR “Sympathetic nervous system” OR “Parasympathetic activity” OR “Sympathetic activity” OR “Cardiac autonomic function” OR “Heart period variability” OR “Cardiac autonomic regulation” OR “Vagal tone” OR “Sympathovagal balance” OR “Autonomic imbalance” OR “Heart rate” OR “R-R interval”) AND ("Wearable Electronic Devices" OR Wearables OR "Smart band" OR "Heart rate monitor" OR "HR monitor" OR "Smart watch" OR Wristband OR "Polar" OR "Garmin" OR "Oura" OR PPG OR Photoplethysmography OR "chest strap" OR "heart rate sensor" OR "Apple Watch" or Fitbit OR Whoop)

Embase

‘female’ AND ('menstrual cycle' OR 'sex hormone' OR ‘ovarian hormone’ OR 'reproductive hormone' OR 'follicular phase' OR 'luteal phase' OR ‘ovulatory phase’ OR 'menstruation' OR 'ovulation' OR ‘ovulate’ OR 'menstruation' OR 'menarche' OR 'fertility' OR 'menopause' OR 'oral contraceptive agent' OR 'hormonal contraceptive agent' OR 'estrogen' OR 'progesterone' OR 'luteinizing hormone release') AND ('heart rate variability' OR 'autonomic nervous system' OR 'cholinergic system' OR 'adrenergic system' OR 'parasympathetic tone' OR 'sympathetic tone' OR 'cardiac autonomic function' OR 'cardiac autonomic regulation' OR 'vagus tone' OR 'sympathovagal balance' OR ‘autonomic balance’ OR 'heart rate' OR 'R- R interval') AND ('wearable computer' OR 'wearable device' OR 'smart band' OR 'heart rate monitor' OR 'heart rate monitoring' OR 'smart watch' OR 'wristband' OR 'polar' OR 'garmin' OR 'oura ring' OR 'photoelectric plethysmography' OR 'chest strap' OR 'heart rate sensor' OR 'apple watch')

PRISMA flowchart depicting the flow of information through the different phases of the systematic review process.

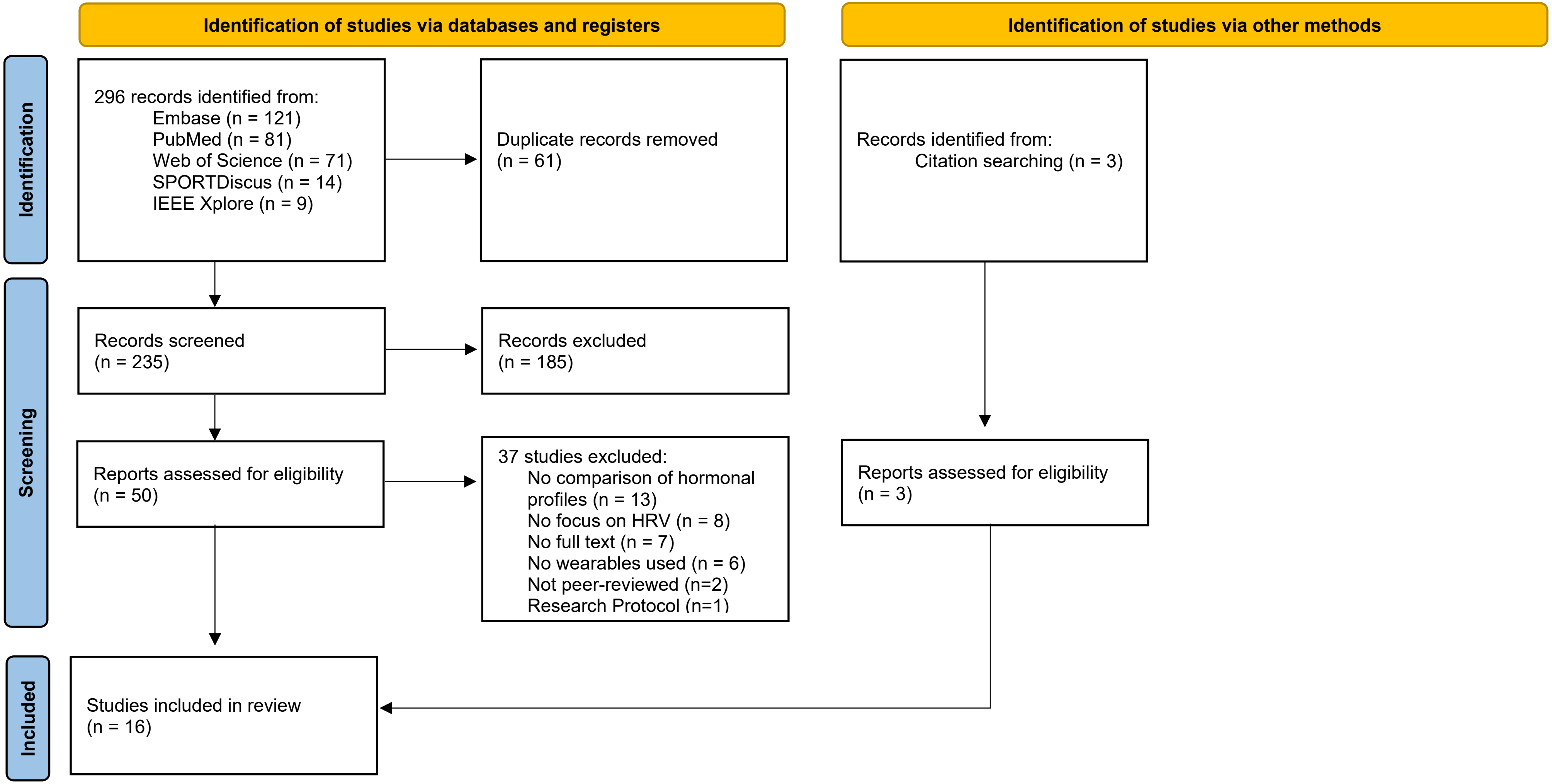

Overview of methods used for classification of menstrual cycle phases and menopausal status

Author (year of publication)	Classification	Methods	Phases	Cycles	Notes
Ahokas et al. (2023) [23]	Menstrual cycle phases	Self-reported menses via MC/HC diary Urinary ovulation kit (Sofi)	**Bleeding =** reported menses<br>**Follicular =** bleeding until ovulation<br>**Ovulatory =** positive test + 7 days<br>**Luteal =** 7 days post OV until menses	one menstrual cycle	Four blood samples were taken to analyse serum hormone concentrations, not for confirmation of phases
	Combined Hormonal contraceptive phases	Self-reported menses via MC/HC diary	**Inactive =** week not taking pill<br>**Active =** weeks taking pill	4 weeks	No confirmed ovulation
	Hormonal contraceptive phases	Not given	**M1 =** lowest E2<br>**M2 =** +7 days<br>**M3 =** +7 days<br>**M4 =** + 7 days	4 weeks	No specification of how lowest E2 is measured
Altini et al. (2021) [24]	Menstrual cycle phases	Self-reported menses	**Follicular =** reported menses until calculated half-way point<br>**Luteal =** half-way point until next menses	At least 5 menstrual cycles	No confirmed ovulation
Alzueta et al. (2022) [25]	Menstrual cycle phases	Self-reported menses in daily survey<br>Commercial urine tests (PREGMATE)	**Menses =** reported menses<br>**Ovulation =** positive test + 1 day<br>**Midluteal =** 6-10 days post OV<br>**Late luteal =** 4 days before next menses	One menstrual cycle	

Alzueta et al. (2024) [26]	Menstrual cycle phases	Self-reported menses in daily diary<br><br>commercial urine test (PREGMATE)<br><br>Oscillations of temperature	**Menses =** reported menses<br><br>**Ovulation** = positive test + 1 day<br><br>**Mid-luteal =** 6-10 days post OV<br><br>**Late-luteal =** 4 days before next menses	At least one menstrual cycle	Cosinor curve fitted on temperature data, $r^2 >$ 0.25, the data were considered to be oscillatory, and therefore reflect an ovulatory menstrual cycle
	Menopausal status	Age	**Young =** 18-35 years<br><br>**Midlife =** 42-55 years		No amenorrhea status confirmed
Andric et al. (2021) [27]	Menstrual cycle phases	Self-reported menses, 14th day was taken as ovulation day	**Early follicular =** day 1-8<br><br>**Late follicular =** day 9-13<br><br>**Mid-luteal =** day 19-23		No confirmed ovulation
Goodale et al. (2019) [28]	Menstrual cycle phases	Self-reported menses<br><br>Testing kit for ovulation (ClearBlue Advanced)	**Menstruation =** self-reported menses<br><br>**Follicular =** after bleeding until 6 days pre-OV<br><br>**Fertile window =** 5 days pre-OV until positive test<br><br>**Early luteal** = OV + 7 days<br><br>**Late luteal** = OV + 8 days until next menses	Up to a year or until pregnant	
Gordon et al. (2023) [29]	Menstrual cycle phases	Self-reported menses andbasal-body temperature into tracking app	**Low hormones =** day 2-8<br><br>**High hormones =** day 14-18		NM and CHC combined

		Salivary oestrogen used to determine phases			
	Combined Hormonal contraceptive phases	Salivary oestrogen used to determine phases	**Low hormones =** inactive pill week<br><br>**High hormones =** active pill weeks		
Hamidovic et al. (2023) [30]	Menstrual cycle phases	Self-reported menses<br><br>Urine tests (Clearblue)<br><br>Blood sample for DHEA and LH for confirmation of LH surge	**Menses =** self-reported menses<br><br>**Mid follicular =** day 5-8<br><br>**Peri-ovulatory =** 2 days around positive test<br><br>**Luteal =** 5 days post OV until next menses	One menstrual cycle	Birth control users combined with NM
Jasinski et al. (2024) [31]	Menstrual cycle phases	Self-reported menses in daily survey	**Menses =** reported bleeding<br><br>**Late luteal =** days before next menses	95% days of wear across minimum of two cycles	No confirmed ovulation
	Combined Hormonal contraceptive phases	Self-reported menses in daily survey	**Menses =** reported bleeding<br><br>**Late luteal =** days before next menses	95% days of wear across minimum of two cycles	
Kokts-Porietis et al. (2019) [32]	Menstrual cycle phases	Basal body temperature taken by Geratherm digital thermometer	**Follicular =** days before temperature rise peak<br><br>**Luteal =** days after peak	At least five consecutive weeks	Sensiplan method of defining BBT rise to signal ovulation used
Luo et al. (2025) [33]	Menstrual cycle phases	Self-reported menses through app	**Follicular =** Menses until 6 days pre-OV<br><br>**Fertile phase =** 6 days pre-OV until OV	Two complete menstrual cycles with <80% data	Determination of ovulation based on follicular diameter,

		Follicular ultrasounds<br><br>Blood tests for E2, P4, and LH	**Luteal phase =** OV until next menses		hormone concentrations and ultrasound confirmation of follicular rupture
Markovic et al. (2024) [34]	Menopausal status	Self-reported menses	**Not menstruating =** pregnant and post-menopausal (description not given)<br><br>**Menstruating =** menstruation occurs<br><br>**Perimenopausal =** description not given	Up to nine months	No amenorrhea status confirmed
	Menstrual cycle phases	Wearable algorithm based on physiological signals	**Menses** = 5 days from first day of menses<br><br>**Follicular** = From the first day post menses until 6 days pre-OV<br><br>**Fertile window** = 5 days pre-OV until OV<br><br>**Early luteal** = Day after OV + 7 days<br><br>**Late luteal** = OV + 8 days until menses	Up to nine months	No confirmed ovulation
Pearson et al. (2025) [35]	Menstrual cycle phases	Self-reported menses via REDCap<br><br>Dual hormone ovulation kits (Clearblue)	**Follicular =** days before OV<br><br>**Luteal =** days after OV	16 weeks	Oestradiol and progesterone were assessed from blood samples at three times, not for confirmation of phases
Sanchez-Barajas et al. (2018) [36]	Menopausal status	STRAW criteria used for menopausal stage confirmation	**Pre-menopausal =** regular menstrual periods<br><br>**Early postmenopausal =** aged 45-57 and <5 years since last menses		FSH and cortisol were measured by ELISA commercial kits (ALPCO)

			**Late postmenopausal =** aged 57+ and >5 years since last menses		
Sherman et al. (2021) [37]	Menstrual cycle phases	Self-reported menses in survey	**Menses =** reported menses<br><br>**No menses =** no reported menses	18 weeks	NM and CHC combined
Sims et al. (2021) [38]	Menstrual cycle phases	Self-reported menses	**Early follicular =** 1-25% of cycle<br><br>**Late follicular =** 26-50% of cycle<br><br>**Early luteal =** 51-75% of cycle<br><br>**Late luteal =** 76-100% of cycle		No confirmed ovulation
	Combined Hormonal contraceptive phases	Self-reported menses	**Week 1 =** self-reported menses<br><br>**Week 2-4 =** active pill weeks		
	Hormonal contraceptive phases	Self-reported menses	**Week 1 =** self-reported menses<br><br>**Week 2-4 =** active pill weeks		

LH: Luteinizing hormone, OV: ovulation, E2: oestrogen, NM: naturally menstruating, CHC: combined hormonal contraceptive users, DHEA: dehydroepiandrosterone, P4: progesterone

Risk of bias table

Results from the risk of bias assessment done with the Newcastle-Ottawa Assessment Scale for cohort studies

	Selection	Comparability	Outcome	Total Score
Ahokas et al. (2023)	★★★	★★	★★	7
Altini et al. (2021)	★★	★★	★★	6
Alzueta et al. (2022)	★★★	★★	★	6
Alzueta et al. (2024)	★★★★	★★	★	7
Andric et al. (2021)	★★★	★★	★★	7
Goodale et al. (2019)	★★★	★★	★★★	8
Gordon et al. (2023)	★★★★	★★	★	7
Hamidovic et al. (2023)	★★★	★★	★★	7
Jasinski et al. (2024)	★★	★★	★★★	7
Kokts-Porietis et al. (2020)	★★★	★★	★★	7
Luo et al. (2025)	★★★	★★	★★	7
Markovic et al. (2024)	★★★★	★★	★★★	9
Pearson et al. (2025)	★★★★	★★	★	7
Sanchez-Barajas et al. (2018)	★★★	★★	★	6
Sherman et al. (2022)	★★★	★★	★★	7
Sims et al. (2021)	★★★	★★	★	6
